# Exploring Valid Reference Genes for Quantitative Real-Time PCR Analysis in *Sesamia inferens* (Lepidoptera: Noctuidae)

**DOI:** 10.1371/journal.pone.0115979

**Published:** 2015-01-13

**Authors:** Meng Sun, Ming-Xing Lu, Xiao-Tian Tang, Yu-Zhou Du

**Affiliations:** 1 School of Horticulture and Plant Protection & Institute of Applied Entomology, Yangzhou University, Yangzhou, Jiangsu, China; 2 Institute of Plant Protection, Shangdong Academy of Agricultural Sciences, Jinan, Shandong, China; Institute of Vegetables and Flowers, Chinese Academy of Agricultural Science, CHINA

## Abstract

The pink stem borer, *Sesamia inferens*, which is endemic in China and other parts of Asia, is a major pest of rice and causes significant yield loss in this host plant. Very few studies have addressed gene expression in *S. inferens*. Quantitative real-time PCR (qRT-PCR) is currently the most accurate and sensitive method for gene expression analysis. In qRT-PCR, data are normalized using reference genes, which help control for internal differences and reduce error between samples. In this study, seven candidate reference genes, 18S ribosomal RNA (*18S rRNA*), elongation factor 1 (*EF1*), glyceraldehyde-3-phosphate dehydrogenase (*GAPDH*), ribosomal protein S13 (*RPS13*), ribosomal protein S20 (*RPS20*), tubulin (*TUB*), and β-actin (*ACTB*) were evaluated for their suitability in normalizing gene expression under different experimental conditions. The results indicated that three genes (*RPS13*, *RPS20*, and *EF1*) were optimal for normalizing gene expression in different insect tissues (head, epidermis, fat body, foregut, midgut, hindgut, Malpighian tubules, haemocytes, and salivary glands). *18S rRNA*, *EF1*, and *GAPDH* were best for normalizing expression with respect to developmental stages and sex (egg masses; first, second, third, fourth, fifth, and sixth instar larvae; male and female pupae; and one-day-old male and female adults). *18S rRNA*, *RPS20*, and *TUB* were optimal for fifth instars exposed to different temperatures (−8, −6, −4, −2, 0, and 27°C). To validate this recommendation, the expression profile of a target gene heat shock protein 83 gene (*hsp83*) was investigated, and results showed the selection was necessary and effective. In conclusion, this study describes reference gene sets that can be used to accurately measure gene expression in *S. inferens*.

## Introduction

Gene expression analysis is increasingly important in insect molecular biology. Quantitative real-time PCR (qRT-PCR) is possibly the best method to analyze gene expression because of the large dynamic range and high sensitivity and reproducibility [1–5]. However, variations in RNA extraction, reverse transcription, cDNA concentration, and PCR efficiency make qRT-PCR analysis prone to error [6,7]. To obtain reliable and valid gene expression profiles, controls are essential for normalizing expression [8]. A variety of “housekeeping” genes have been utilized to normalize gene expression [9]. An ideal reference gene should exhibit similar, stable mRNA expression levels under different biotic and abiotic conditions. Housekeeping genes are involved in basic, ubiquitous cellular functions. Examples include the genes encoding β-actin (*ACTB*), glyceraldehyde-3-phosphate dehydrogenase (*GAPDH*) and 18S ribosomal RNA (*18S rRNA*); these genes have been used extensively to normalize expression in many different organisms [1–4]. The assumption is that these housekeeping genes are uniformly expressed regardless of the experimental conditions. However, previous studies have demonstrated that these widely-used reference genes are differentially expressed under various experimental conditions [10–12], which has reduced their utility in gene expression analysis [13]. Normalization with unstable internal controls may result in different values and lead to erroneous results. Thus, it is necessary to meticulously evaluate the expression profiles of candidate reference genes for each experimental system. The comparative ΔCt method [14] and various computational programs (NormFinder [15], geNorm [16], BestKeeper [17]) have been used to identify the best-suited reference genes for various organisms. The web-based tool, RefFinder, integrates these computational programs to compare candidate reference genes [18].

The pink stem borer, *Sesamia inferens* (Walker) (Lepidoptera: Noctuidae), is a major pest of rice in China and other Asian countries. Recently, damage incited by *S. inferens* has become more serious. In addition, based on our investigation, the pest now occurs in more northern regions of China (unpublished data). Many studies of *S. inferens* have focused on biological characteristics [19–23]. However, the underlying mechanisms that explain the outbreak and distribution of *S. inferens* remain obscure. Expression analysis of relevant genes may provide insight on the incidence of *S. inferens*; however, effective and credible reference gene combinations are needed for *S. inferens* before this approach is undertaken.

In this study, seven candidate reference genes of *S. inferens* including *18S rRNA*, elongation factor 1 (*EF1*), *GAPDH*, ribosomal protein S13 (*RPS13*), ribosomal protein S20 (*RPS20*), tubulin (*TUB*), and *ACTB* were evaluated for their suitability in the normalization of gene expression under various experimental conditions (different tissues, developmental stages, sex, and temperature). The aim of this work was to address an important and often neglected aspect of gene expression insects: e.g. the validation of appropriate reference genes with stable expression levels in different sample groups. To further validate our results, the expression profile of the gene encoding heat shock protein 83 (*hsp83*), a member of the *hsp90* gene family [24], was investigated. To our knowledge, this study is the first to document the stability of reference gene expression in *S. inferens*.

## Materials and Methods

### Insects

*S. inferens* populations were collected from Jiangdu (32°39′ N, 119°42′ E), which is located in Yangzhou, Jiangsu province. In this paper, all study sites are public lands. Our research activities were not banned by any organization or individual, and did not involve endangered or protected species.

The pink stem borers were reared in an environmental chamber at 27 ± 1°C with a 16:8 (light/dark) photoperiod and 60–70% relative humidity as described previously [25]. In order to make the results more accurate, age of *S. inferens* larvae was synchronized before the experiment.

### Tissues

In this part of the study, different tissues of fifth instar larvae were analyzed. The larvae selected were similar in size and were randomly assigned to experimental groups. The larvae had been starved for 24 h before dissection to evacuate the digestion tract. Each group contained ten larvae, and each experiment was repeated three times. Larvae were anesthetized on ice before dissection. The head, epidermis, fat body, foregut, midgut, hindgut, Malpighian tubules, haemocytes, and salivary glands were collected from larvae of same replicate and rinsed with a 0.9% sodium chloride solution. The samples were frozen immediately in liquid nitrogen and stored at −70°C prior to analysis.

### Developmental stage and sex

This experiment focused on different developmental stages and sexes. Samples included egg masses, the first, second, third, fourth, fifth and sixth instar larvae, male and female pupae, and one-day-old male and female adults; these were randomly selected for the experiment. Each group contained ten insects at least, and each experiment was repeated three times. The samples were frozen immediately in liquid nitrogen and stored at −70°C until needed for analysis.

### Cold tolerance

In this experiment, larvae (*n* ≥ 10) of fifth instar were placed individually in glass tubes, and groups of ten were then exposed to various temperatures (−8, −6, −4, −2 and 0°C) for 2 h in a constant-temperature incubator (DC-3010, Jiangnan Equipment). The larvae were recovered at 27 ± 1°C for 2 h, after which surviving larvae were frozen in liquid nitrogen and stored at −70°C. A set of larvae maintained at 27 ± 1°C was regarded as a control group. Each treatment was repeated three times, and included at least three surviving larvae.

### Quantitative real-time PCR

Total RNA was extracted using the SV Total RNA isolation system (Promega, USA), followed by DNase treatment. The integrity of RNA in all samples was verified by comparing the rRNA bands in ethidium bromide-stained gels. RNA sample purity was estimated by spectrophotometric measurement at 260 and 280 nm (Eppendorf BioPhotometer plus). To ensure consistent amounts of cDNA, we measured the concentration of RNA twice for each sample. RNA (0.5 μg) was reverse-transcribed into first-strand cDNA using the Bio-Rad iScript cDNA Synthesis Kit. Real-time PCR reactions were performed in a 20 μl total reaction volume comprised of 10 μl Bio-Rad iTaq Universal SYBR Green supermix (2×), 1 μl of each gene-specific primer (10 μM) (Table 1), 2 μl of cDNA templates (10× dilution), and 6 μl of H_2_O. Reactions were carried out on a Bio-Rad CFX-96 real-time PCR system under the following conditions:3 min of polymerase activation at 95°C; followed by 40 cycles of denaturation at 95°C for 30 s, and annealing at the Tm for each gene (30 s; Table 1). Each treatment included three replicates, and each reaction was run in triplicate.

**Table 1:** Primers used for qRT-PCR analysis.

**Gene**	**Primer sequences (5’-3’)**	**Amplicon size**	**PCR efficiency**	**Tm**	**R^2^**
*18S rRNA*	F:CAACACGGGAAATCTCACCA	115 bp	107.3%	55.6°C	0.996
	R:GACAAATCGCTCCACCAACTAA				
*EF1*	F:GTCGCTTTCGTACCCATTTCT	86 bp	97.4%	56.6°C	0.994
	R:ACAGTCCATCCCTTGAACCAG				
*GAPDH*	F:GGTCATCTCCAACGCTTCCT	166 bp	95.0%	56.6°C	0.993
	R:ACGTCCATCACGCCACAAT				
*RPS13*	F:TGGTAAGGGTATCTCCCAATCA	75 bp	93.5%	60.1°C	0.994
	R:TCGTCAGCAGTCAGTTTCAGC				
*RPS20*	F:CTCATCAATGGAGCCAAGAAAC	162 bp	102.0%	60.1°C	0.986
	R:GTGCAGGTCAATGACACGCT				
*TUB*	F:TTGCTACAGAACCCTCAAAGTGC	159 bp	104.4%	59.2°C	0.985
	R:AGACGTGGGAACGGAACCAT				
*ACTB*	F:ATGGTTGGTATGGGTCAGAAGG	191 bp	95.2%	62.1°C	0.985
	R:TCAGTCAGGAGGACTGGGTGTT				
*hsp83*	F: GTCTGCCAACATGGAGCGTA	167 bp	102.5%	57.5°C	0.997
	R: GGTCCTTCACAGCCTTATCGT				

### Data analysis

Data were analyzed using the Bio-Rad CFX Manager 3.1 software. The cycle threshold (Ct value) denotes the cycle at which the fluorescent signal first shows significant difference with respect to the background. All biological replicates were used to calculate the average Ct value. Stability of the candidate reference genes was evaluated using the comparative ΔCt method [14] and three software tools (NormFinder [15], geNorm [16] and BestKeeper [17]). The comparative ΔCt method was used to compare relative expression of pairs of genes within each sample. NormFinder ranks the stability of each candidate gene independently. The geNorm algorithm calculates an expression stability value (M) for each gene and then performs a pair-wise comparison (Vn/Vn+1) of this gene with others. BestKeeper determines the standard deviation with the user selecting the best genes based on these variables. Finally, we compared and ranked the candidate genes based on the web-based analysis tool, RefFinder [18]. Using the rankings from each program, RefFinder assigns an appropriate weight to an individual gene and calculates the geometric mean of their weights for the overall final ranking.

### Validation of reference gene selection

The target gene *hsp83* was used to assess the validity of selected reference genes. *hsp83* expression was determined in *S. inferens* tissues, developmental stages, sexes, and temperatures with gene-specific primers (Table 1).We used the recommended reference genes based on qRT-PCR results. The least stable gene (as determined by RefFinder) was used for comparative purposes. Relative expression of *hsp83* was calculated using the 2^−ΔΔCt^ method [26]. Tukey’s test was conducted for statistical analysis using PASW Statistics 18.0.

## Results

### Transcriptional profiling of candidate reference genes

Expression of the eight genes (seven potential reference genes and *hsp83*) was investigated by reverse transcription polymerase chain reaction (RT-PCR). All genes were expressed in *S. inferens* and were visualized as single bands of the expected size on 1% agarose gels. All amplicons were sequenced and displayed 91–100% identity with corresponding gene sequences. Furthermore, the presence of a single, sharply defined peak in melting curve analysis was confirmed (S1 Fig.). A standard curve was generated for each gene using a serial dilution (1×, 10×, 10^2^×, 10^3^×, 10^4^×, 10^5^×, 10^6^× and 10^7^×) of the pooled cDNAs. The PCR efficiency (calculated from the standard curve) and correlation coefficient (R^2^) for each standard curve are shown in Table 1.

Expression levels were determined as the number of cycles needed for amplification to reach a fixed threshold in the exponential phase of the qRT-PCR reaction. As shown in Table 2, seven candidate reference genes exhibited relatively different variation in Ct values.

**Table 2:** Cycle threshold of candidate reference genes in experimental conditions.

**Conditions**		**Cycle threshold (Ct value, mean ± SE)**						
		***18S rRNA***	***EF1***	***GAPDH***	***RPS13***	***RPS20***	***TUB***	***ACTB***
**Tissues**	HE	12.40 ± 0.30	24.13 ± 0.44	23.61 ± 0.30	19.83 ± 0.18	22.22 ± 0.15	26.93 ± 0.26	23.71 ± 0.47
	EP	11.32 ± 0.46	21.86 ± 0.27	22.09 ± 0.37	19.60 ± 0.12	21.77 ± 0.15	26.46 ± 0.38	21.29 ± 0.57
	FG	13.49 ± 0.14	24.25 ± 0.13	23.45 ± 0.16	20.39 ± 0.14	23.05 ± 0.16	28.46 ± 0.27	28.77 ± 0.13
	MG	12.68 ± 0.34	23.16 ± 0.18	24.06 ± 0.19	20.32 ± 0.12	23.17 ± 0.08	27.24 ± 0.26	27.52 ± 0.28
	HG	13.90 ± 0.50	23.42 ± 0.42	22.34 ± 0.19	20.40 ± 0.25	22.14 ± 0.16	26.81 ± 0.37	24.68 ± 0.34
	HC	13.58 ± 0.34	23.79 ± 0.41	25.06 ± 0.26	20.76 ± 0.21	23.00 ± 0.08	27.97 ± 0.35	35.35 ± 0.68
	FB	12.73 ± 0.28	23.89 ± 0.18	24.41 ± 0.39	21.05 ± 0.15	23.25 ± 0.11	28.76 ± 0.43	31.83 ± 0.60
	MT	14.91 ± 0.22	24.63 ± 0.22	22.27 ± 0.35	20.93 ± 0.21	22.85 ± 0.18	28.39 ± 0.26	29.90 ± 0.23
	SG	14.15 ± 0.26	26.25 ± 0.61	29.58 ± 0.37	22.42 ± 0.17	24.02 ± 0.17	28.41 ± 0.31	33.47 ± 0.73
**Developmental stages and sex**	E	12.29 ± 0.15	23.11 ± 0.07	22.48 ± 0.14	21.45 ± 0.09	32.08 ± 0.18	26.46 ± 0.13	38.76 ± 0.20
	L1	13.76 ± 0.14	25.50 ± 0.35	24.50 ± 0.31	25.99 ± 1.24	24.91 ± 0.24	32.99 ± 0.19	26.80 ± 0.31
	L2	13.81 ± 0.57	24.60 ± 0.50	24.81 ± 0.52	28.07 ± 0.42	24.90 ± 0.34	29.50 ± 0,24	29.30 ± 0.52
	L3	11.87 ± 0.07	24.33 ± 0.28	24.36 ± 0.13	27.23 ± 0.31	24.62 ± 0.18	29.31 ± 0.19	28.24 ± 0.49
	L4	12.01 ± 0.16	23.78 ± 0.30	24.46 ± 0.36	27.31 ± 0.18	25.16 ± 0.16	28.93 ± 0.18	26.49 ± 0.30
	L5	12.49 ± 0.06	24.70 ± 0.08	24.16 ± 0.19	28.78 ± 0.56	25.88 ± 0.15	29.97 ± 0.17	28.22 ± 0.42
	L6	12.51 ± 0.09	24.47 ± 0.20	25.11 ± 0.27	27.19 ± 0.21	25.39 ± 0.20	29.14 ± 0.57	28.19 ± 0.57
	FP	12.38 ± 0.07	24.80 ± 0.39	25.74 ± 0.06	28.23 ± 0.24	26.03 ± 0.16	27.42 ± 0.10	28.73 ± 0.27
	MP	12.59 ± 0.12	24.78 ± 0.18	26.46 ± 0.21	27.76 ± 0.22	25.21 ± 0.18	28.67 ± 0.54	28.67 ± 0.32
	FA	13.78 ± 0.08	27.07 ± 0.93	24.23 ± 0.14	29.65 ± 0.37	26.03 ± 0.20	31.35 ± 0.19	29.61 ± 0.47
	MA	13.26 ± 0.13	25.56 ± 0.41	23.83 ± 0.11	28.69 ± 0.29	26.41 ± 0.17	30.56 ± 0.13	28.95 ± 0.43
**Temperatures (°C)**	27	12.58 ± 0.52	25.06 ± 0.16	24.24 ± 0.17	27.47 ± 0.33	26.98 ± 1.48	28.54 ± 0.70	26.28 ± 0.94
	0	11.01 ± 0.02	23.98 ± 0.14	24.95 ± 0.10	28.62 ± 0.59	24.74 ± 0.19	26.24 ± 0.02	22.45 ± 0.04
	−2	11.20 ± 0.02	24.71 ± 0.27	25.75 ± 0.07	27.99 ± 0.27	24.40 ± 0.10	25.14 ± 0.02	23.41 ± 0.12
	−4	12.20 ± 0.06	25.11 ± 0.13	25.19 ± 0.34	29.18 ± 0.45	25.40 ± 0.13	25.30 ± 0.04	22.75 ± 0.05
	−6	11.58 ± 0.27	25.97 ± 0.24	25.41 ± 0.07	28.34 ± 0.17	26.32 ± 0.14	26.27 ± 0.20	23.29 ± 0.27
	−8	11.37 ± 0.03	23.51 ± 0.13	26.32 ± 0.16	27.80 ± 0.33	24.20 ± 0.23	25.33 ± 0.05	21.84 ± 0.12

The abbreviations HE, EP, FG, MG, HG, HC, FB, MT, and SG represent heads, epidermis, foregut, midgut, hindgut, haemocytes, fat body, Malpighian tubules, and salivary glands, respectively. And eggs, larvae (first, second, third, fourth, fifth, and sixth instar), female and male pupae, and female and male adults, which are designated E, L1, L2, L3, L4, L5, L6, FP, MP, FA, and MA, respectively.

### Stability of candidate reference genes

#### Tissues

**Figure 1:**
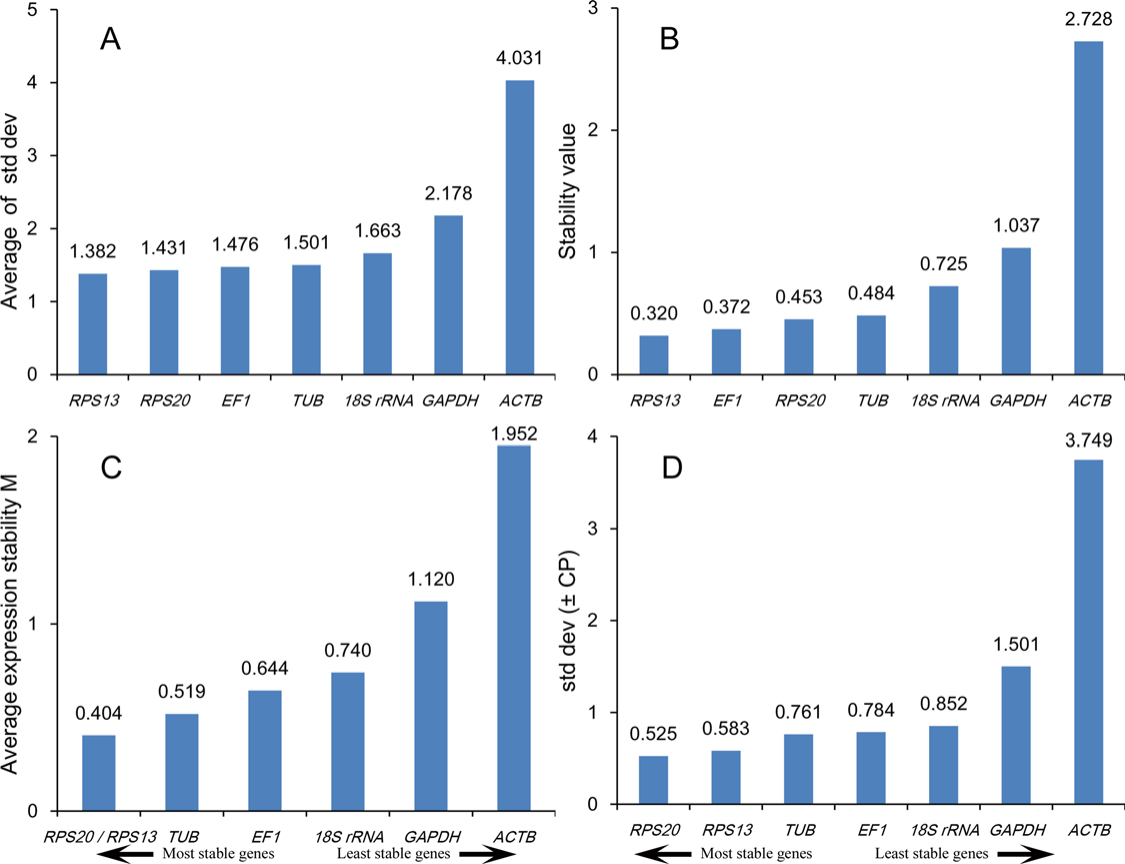
Stability of reference gene expression in different tissue samples. Candidate reference genes (*18S rRNA, EF1, GAPDH, RPS13, RPS20, TUB* and *ACTB*) were evaluated using the comparative ΔCt method (A), NormFinder (B), geNorm (C) and BestKeeper (D).

All analysis methods revealed that *RPS13, RPS20, EF1* and *TUB* were among the four most stable genes. *18S rRNA, GAPDH* and *ACTB* were not appropriate choices for reference genes in different tissue samples (Fig. 1). According to RefFinder, the ranking of most to least stable expression in different tissue samples was as follows: *RPS13* > *RPS20 > EF1 > TUB > 18S rRNA > GAPDH > ACTB* (Table 3).

**Table 3:** Ranking order of the candidate reference genes as determined by RefFinder.

**Conditions**	**Rank**	**Gene**	**Geomean of ranking values**
**Tissues**	1	*RPS13*	1.19
	2	*RPS20*	1.57
	3	*EF1*	3.13
	4	*TUB*	3.46
	5	*18S rRNA*	5.00
	6	*GAPDH*	6.00
	7	*ACTB*	7.00
**Developmental stages and sex**	1	*18S rRNA*	1.19
	2	*EF1*	1.41
	3	*GAPDH*	3.00
	4	*TUB*	4.23
	5	*RPS20*	4.95
	6	*RPS13*	5.73
	7	*ACTB*	7.00
**Temperatures**	1	*18S rRNA*	1.32
	2	*RPS20*	2.21
	3	*TUB*	2.99
	4	*EF1*	3.66
	5	*RPS13*	4.16
	6	*ACTB*	5.63
	7	*GAPDH*	5.66

**Figure 2:**
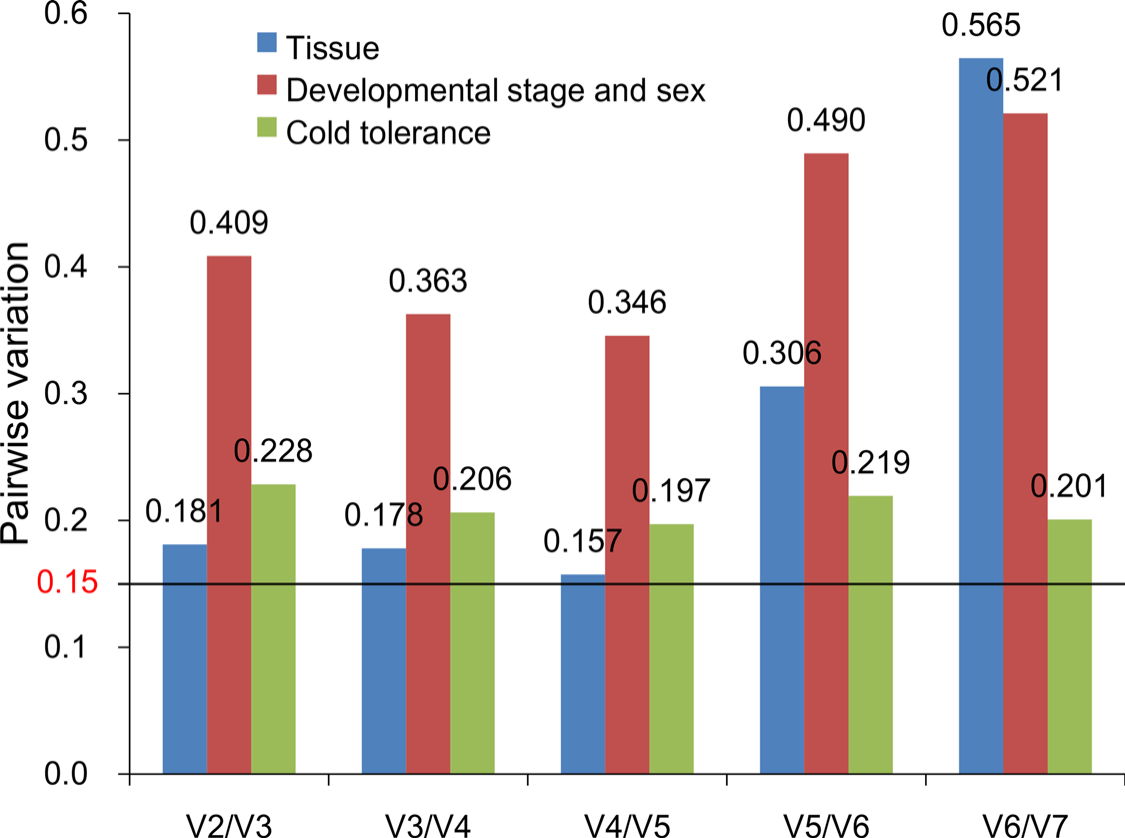
Optimal number of reference genes for normalization in *Sesamia inferens*. The pairwise variation (Vn/Vn+1) was analyzed by the geNorm software to determine the optimal number of reference genes included in the qRT-PCR analysis. Average value of pairwise variations (V) dictates whether inclusion of an extra reference gene would add to the stability of the normalization factor.

geNorm uses 0.15 as a cut-off threshold, and values below this indicate that the inclusion of additional reference genes is unnecessary. The geNorm instruction manual suggests 0.15 as a proposed value, although this cut-off value may need modification according to the experimental parameters and results. It is important to mention that inclusion of the three best reference genes is also a valid normalization strategy, and results in a more accurate and reliable normalization as compared to the use of a single gene. In other words, if all V values were above 0.15, it can be assumed that three reference genes were reliable for normalization. In the different tissue samples, all pairwise (Vn/Vn+1) values were above 0.15 (Fig. 2). Thus, the three reference genes recommended for this subset were *RPS13, RPS20* and *EF1*.

#### Developmental stage and sex

**Figure 3:**
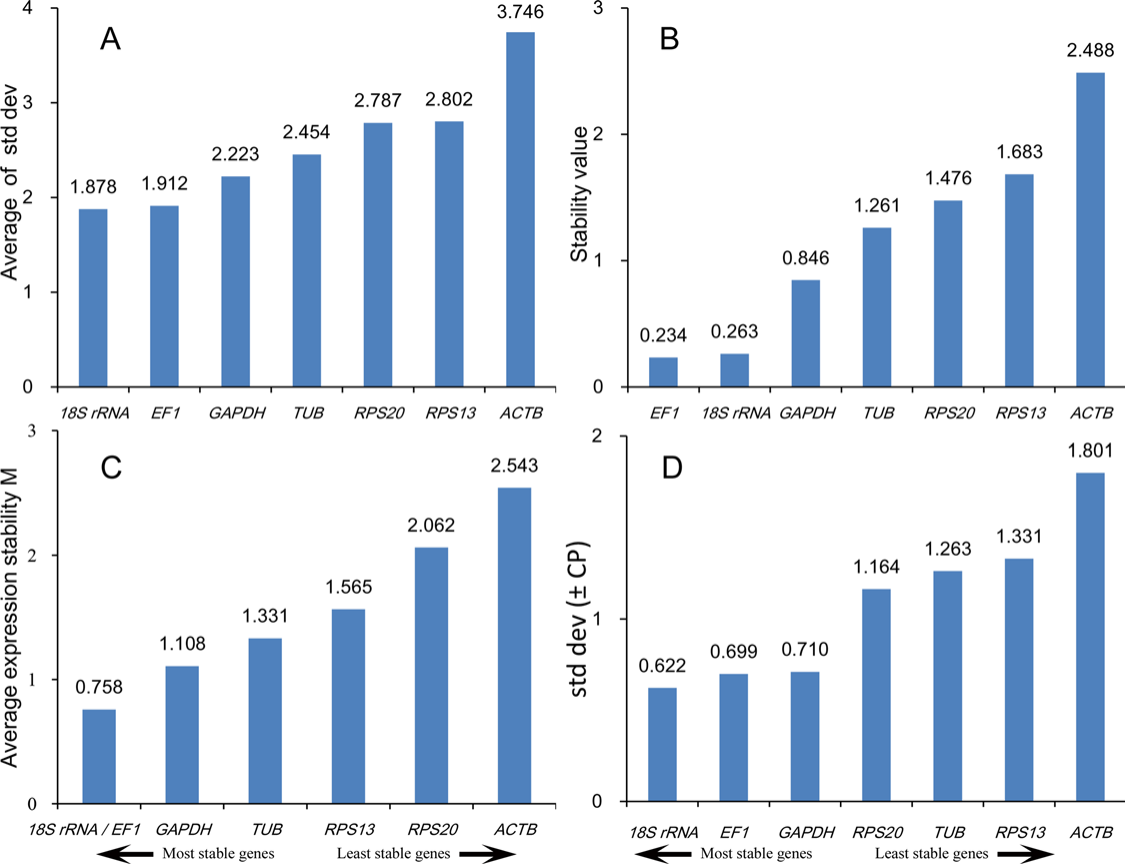
Stability of reference gene expression in different developmental stages and sexes. Candidate reference genes (*18S rRNA, EF1, GAPDH, RPS13, RPS20, TUB* and *ACTB*) were evaluated using the comparative ΔCt method (A), NormFinder (B), geNorm (C) and BestKeeper (D).

With the exception of BestKeeper, the computational programs indicated that *18S rRNA, EF1, GAPDH* and *TUB* were the most stable reference genes in different developmental stages and sexes. Analysis using BestKeeper indicated that *18S rRNA, EF1, GAPDH* and *RPS20* were the most stable reference genes (Fig. 3). Analysis of the data from different developmental stages and sex by RefFinder ranked the order from most to least stable as follows: *18S rRNA > EF1 > GAPDH > TUB > RPS20 > RPS13 > ACTB* (Table 3).

geNorm analysis of expression data from developmental stages and sexes revealed that all pairwise variations (Vn/Vn+1) were above 0.15 (Fig. 2). Thus, the combination of reference genes recommended for this subset was *18S rRNA, EF1* and *GAPDH*.

#### Cold tolerance

**Figure 4:**
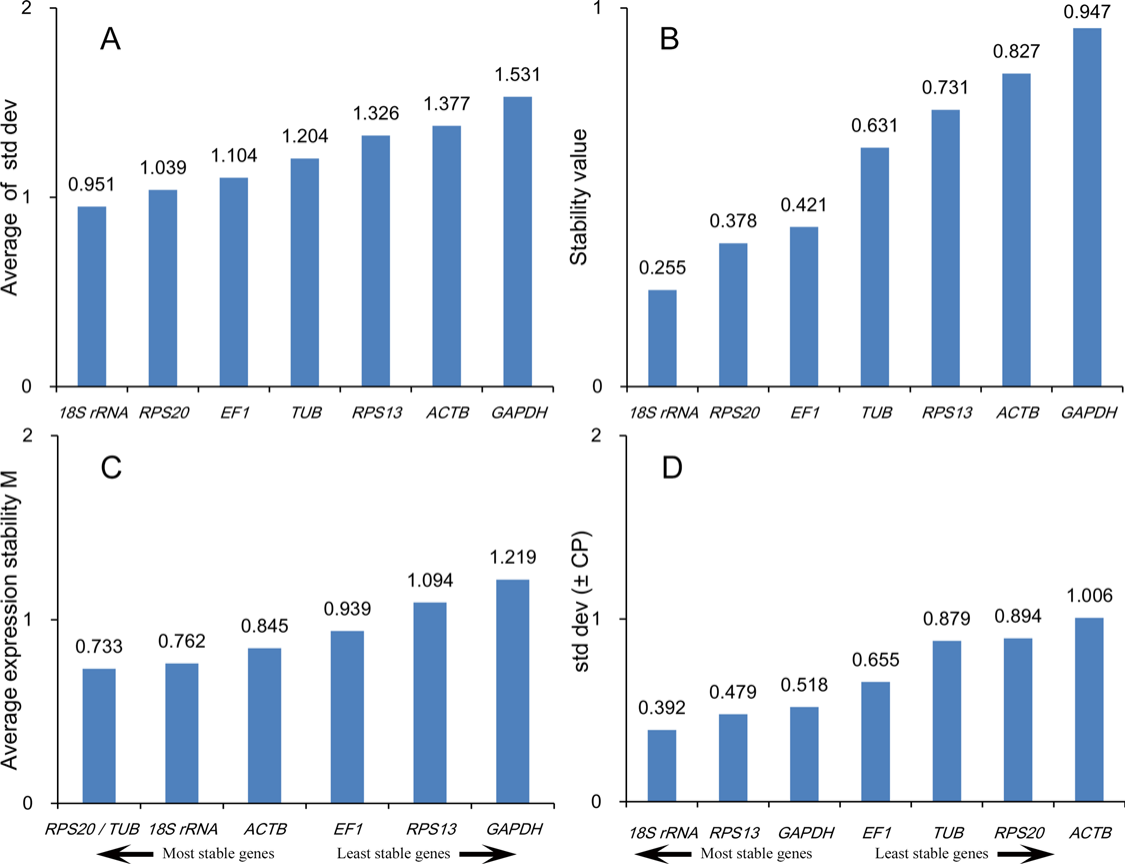
Stability of reference gene expression in samples exposed to cold temperatures. Candidate reference genes (*18S rRNA, EF1, GAPDH, RPS13, RPS20, TUB* and *ACTB*) were evaluated using the comparative ΔCt method (A), NormFinder (B), geNorm (C) and BestKeeper (D).

In samples exposed to cold temperatures, the comparative ΔCt method and NormFinder indicated that *18S rRNA, RPS20, EF1* and *TUB* were the most stable genes. The top four ranked genes analyzed using geNorm were *RPS20, TUB, 18S rRNA* and *ACTB*, and the top four for BestKeeper were *18S rRNA, RPS13, GAPDH* and *EF1* (Fig. 4). According to RefFinder, the overall order from most to least stable in samples exposed to different temperatures was *18S rRNA > RPS20 > TUB > EF1 > RPS13 > ACTB > GAPDH* (Table 3).

geNorm analysis of samples exposed to different temperatures revealed that all pairwise (Vn/Vn+1) values were above 0.15 (Fig. 2). Thus, the combination of reference genes recommended for this subset was *18S rRNA, RPS20* and *TUB*.

### Validation of reference gene selection

#### Tissues

**Figure 5:**
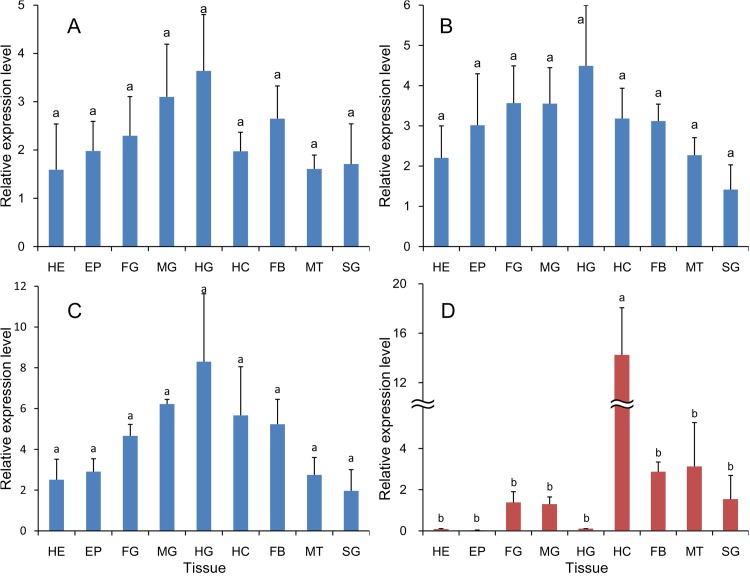
Validation of reference gene selection in tissue samples. The expression level of the target gene *hsp83* was determined tissue samples of fifth instar larvae. The abbreviations HE, EP, FG, MG, HG, HC, FB, MT, and SG represent heads, epidermis, foregut, midgut, hindgut, haemocytes, fat body, Malpighian tubules, and salivary glands, respectively. Three recommended genes (A, *RPS13*; B, *RPS20*; and C, *EF1*) and the least stable gene (D, *ACTB*) were used as reference genes in qRT-PCR. The data shown represent means ± SE. Different letters above bars represent significant difference (*P* = 0.05) (Tukey’s test).

**Figure 6:**
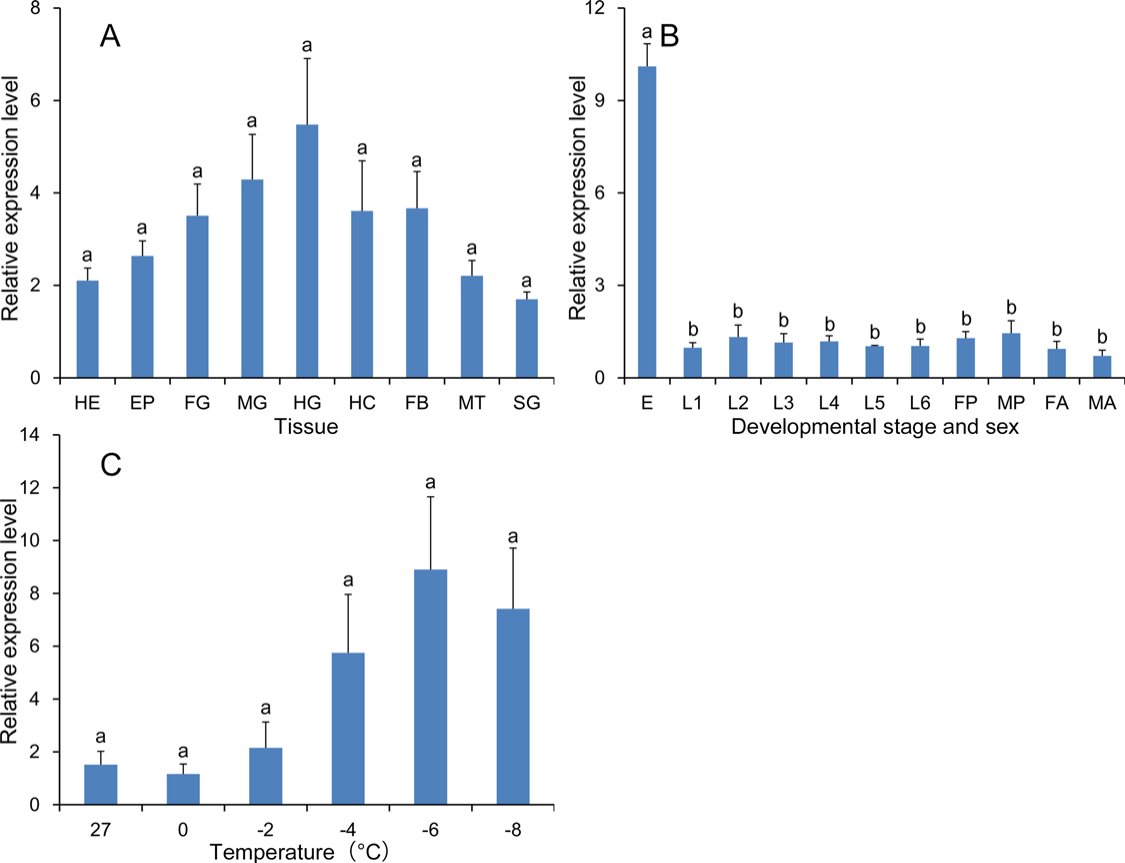
Validation of reference gene selection using 3 reference genes together. The expression level of the target gene *hsp83* was determined at different conditions. Three recommended reference genes were used together. A, *RPS13, RPS20* and *EF1*; B, *18S rRNA, EF1*, and *GAPDH*; C, *18S rRNA, RPS20*, and *TUB*. The abbreviations HE, EP, FG, MG, HG, HC, FB, MT, and SG represent heads, epidermis, foregut, midgut, hindgut, haemocytes, fat body, Malpighian tubules, and salivary glands, respectively. And eggs, larvae (first, second, third, fourth, fifth, and sixth instar), female and male pupae, and female and male adults, which are designated E, L1, L2, L3, L4, L5, L6, FP, MP, FA, and MA, respectively. The data represent the mean ± SE. Different letters above vertical bars represent significant differences (*P* = 0.05) (Tukey’s test).

Relative expression of *hsp83* was calculated using the three recommended reference genes (*RPS13, RPS20* and *EF1*) and the least stable gene (*ACTB*) as an internal control. The expression profiles of *S. inferens hsp83* obtained using the three recommended reference genes were similar, and there were no significant differences between expressions in different tissues (*RPS13*: *F*_8, 18_ = 0.784, *P* = 0.623; *RPS20*: *F*_8, 18_ = 0.984, *P* = 0.479; and *EF1*: *F*_8, 18_ = 1.616, *P* = 0.189) (Fig. 5A-C). Similar expression profiles were obtained when using the three recommended reference genes together as reference (*F*_8, 18_ = 2.332, *P* = 0.065) (Fig. 6A). When *ACTB*, the least stable gene, was used to normalize the data, the expression of *hsp83* was significantly higher in haemocytes than other tissue samples (*F*_8, 17_ = 8.301, *P* < 0.001), and the expression profile of *hsp83* was altered relative to the three recommended genes (Fig. 5D).

#### Developmental stage and sex

**Figure 7:**
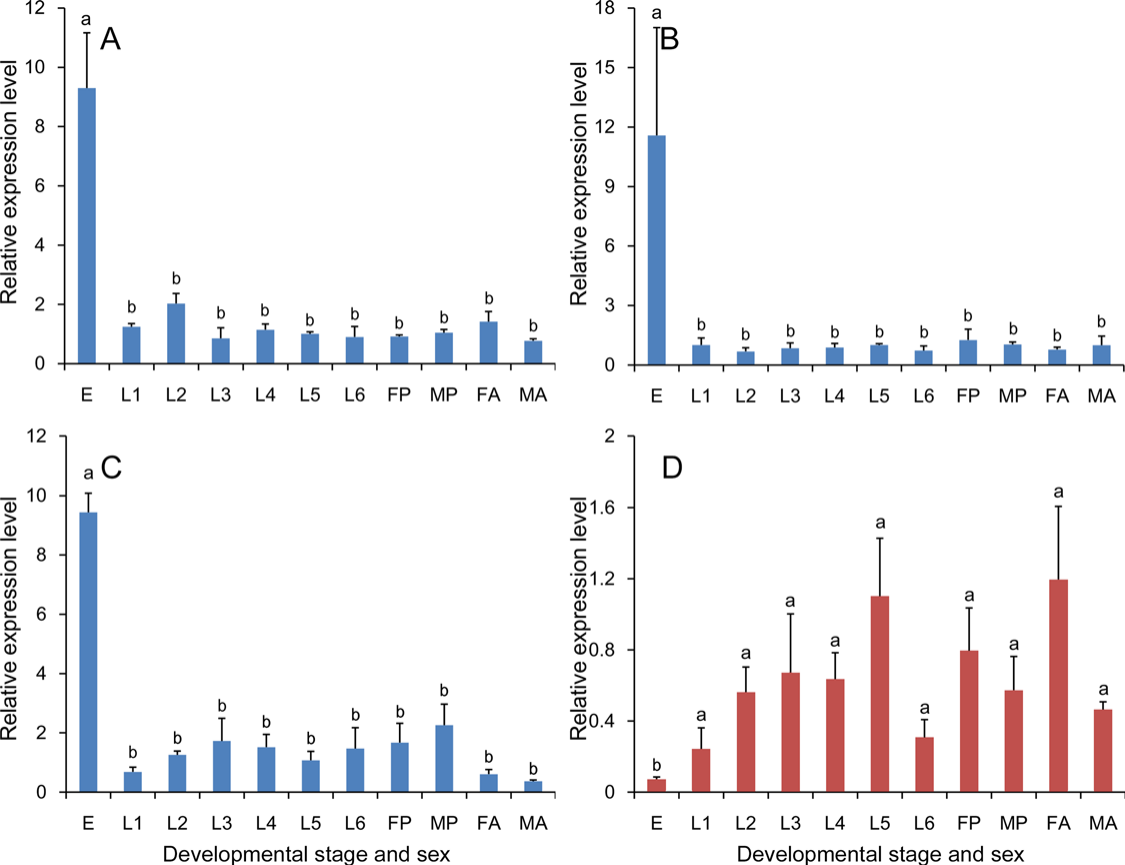
Validation of reference gene selection in samples representing developmental stages and sex. Expression of the target gene *hps83* was determined in eggs, larvae (first, second, third, fourth, fifth, and sixth instar), female and male pupae, and female and male adults, which are designated E, L1, L2, L3, L4, L5, L6, FP, MP, FA, and MA, respectively. Three recommended reference genes (A, *18S rRNA*; B, *EF1*; and C, *GAPDH*) and the least stable gene (D, *ACTB*) were used as reference genes in qRT-PCR. The data are denoted as mean ± SE. Different letters above vertical bars represent significant differences (*P* = 0.05) (Tukey’s test).

Relative expression of *hsp83* in different developmental stages and sexes was calculated using the three recommended reference genes (*18S rRNA, EF1* and *GAPDH*) and one unstable gene (*ACTB*). Similar expression profiles were obtained for *hsp83* when using the *18S rRNA, EF1* and *GAPDH* were used to normalize expression. The expression profiles obtained using the three recommended reference genes together as reference were similar too (*F*_10, 22_ = 68.087, *P* < 0.001) (Fig. 6B). Interestingly, expression in eggs was consistently higher than other tissue samples (*18S rRNA*: *F*_10, 22_ = 16.880, *P* < 0.001; *EF1*: *F*_10, 22_ = 3.738, *P* = 0.005; and *GAPDH*: *F*_10, 22_ = 25.375, *P* < 0.001) (Fig. 7A-C). The expression profile of *hsp83* was altered when the least stable gene, *ACTB*, was used to normalize the data, and expression of *hsp83* in eggs was significant lower than other tissue samples (*F*_10, 22_ = 2.324, *P* = 0.048) (Fig. 7D).

#### Cold tolerance

**Figure 8:**
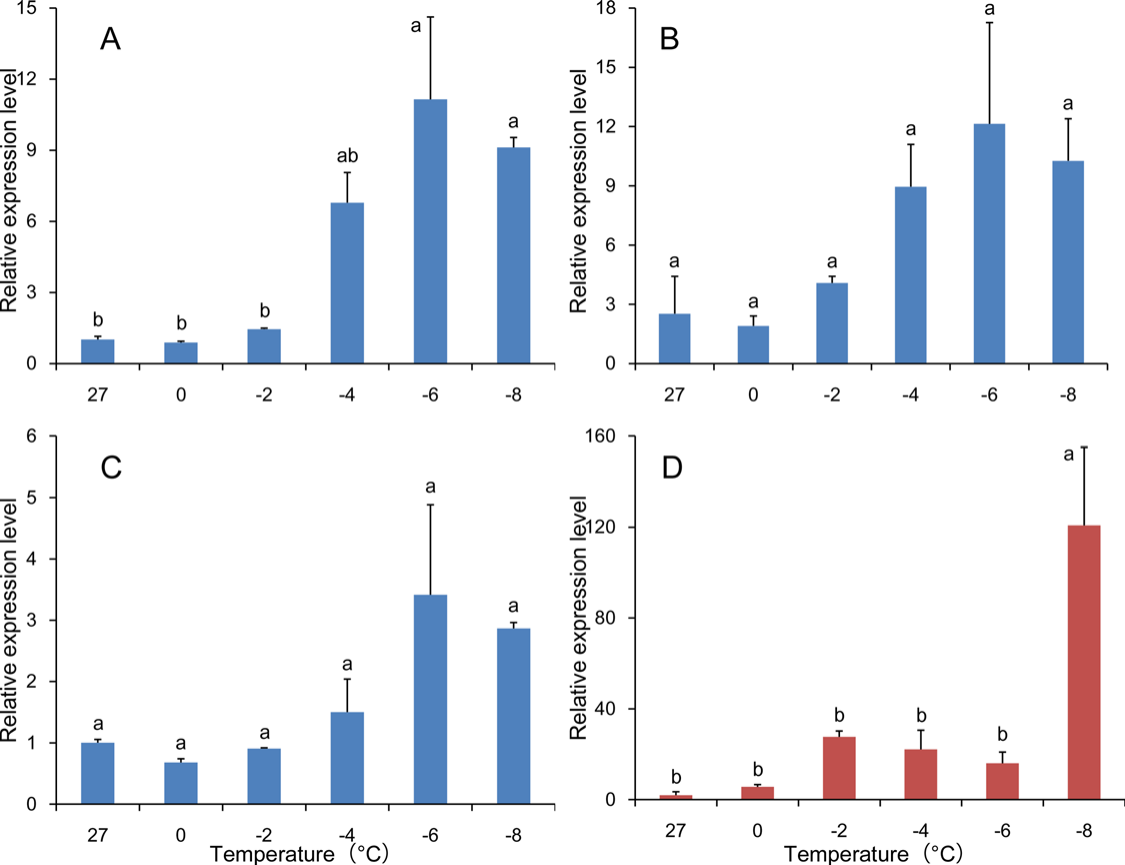
Validation of reference gene selection in different temperature treatments. The expression level of the target gene *hsp83* was determined at different temperature. Three recommended reference genes (A, *18S rRNA*; B, *RPS20*; and C, *TUB*) and the least stable gene (D, *GAPDH*) were used as reference genes in qRT-PCR. The data represent the mean ± SE. Different letters above vertical bars represent significant differences (*P* = 0.05) (Tukey’s test).

The relative expression of *hsp83* in samples subjected to cold treatment was calculated using the three recommended reference genes (*18S rRNA, RPS20* and *TUB*) and the least stable gene (*GAPDH*). When *18S rRNA, RPS20* and *TUB* were used to normalize *hsp83* expression, similar profiles were observed (*18S rRNA*: *F*_5, 12_ = 8.908, *P* = 0.001; *RPS20*: *F*_5, 11_ = 2.591, *P* = 0.087; and *TUB*: *F*_5, 12_ = 3.168, *P* = 0.047) (Fig. 8A-C). The expression profiles obtained using the three recommended reference genes together as reference were similar too (*F*_5, 12_ = 3.451, *P* = 0.037) (Fig. 6C). When *GAPDH* was used to normalize the data, the expression profile of *hsp83* was altered relative to the three recommended genes. An example is the significantly higher expression of *hsp83* in samples treated at −8°C and normalized with *GAPDH* relative to other temperatures (F_5, 12_ = 9.197, *P* = 0.001) (Fig. 8D).

## Discussion

qRT-PCR is a powerful technique for analyzing gene expression. When compared to conventional PCR, qRT-PCR provides a dynamic range of absolute and relative quantification, along with precision, technical sensitivity, and lower risk of sample contamination [1–3]. In qRT-PCR, gene expression levels must be normalized to endogenous reference genes since high variability has been noted. Most gene expression studies in the literature use a single internal control for normalization; thus, the validity of the results relies largely on the control gene used [10]. Therefore, the genes generally used to normalize qRT-PCR in insects should be considered “putative” reference genes [11]. Examples include *ACTB* for *Spodoptera exigua* [27] and the *Bemisia tabaci* ZHJ2 biotype [28] and *18S rRNA* for *Myzus persicae* [29].

Recently, increasing numbers of entomologists have decided that the use of only one or two reference genes for normalizing qRT-PCR data is not acceptable. Consequently, studies designed to explore what reference genes are valid for qRT-PCR analysis in insects have become extremely important. The following criteria should be considered in selecting a reliable reference gene: (1) the gene(s) should have amplification efficiency similar to the target genes; (2) the gene(s) should have a moderate level of gene expression; and (3) expression should be stable in all test samples examined.

Several studies on reference gene selection for insects have been conducted. Ribosomal protein L10 (*RPL10*), arginine kinase (*AK*) and *EF1* were ranked highly as reference genes for *Spodoptera litura* [30]. In *Plutella xylostella, EF1* was the most appropriate reference gene for different insect tissues [31]. In *Spodoptera exigua*, ribosomal protein L10 (*RPL10*), elongation factor 2 (*EF2*) and ribosomal protein L17A (*RPL17A*) performed well in all tissues types [32]. In our study, we show that *RPS13, RPS20* and *EF1* comprise the best set of reference genes for *S. inferens*.

In different developmental stages of *Liposcelis bostsrychophila*, reference genes could be ranked from the most to least stable as follows *Lbβ-Actin1* > *Lbα-Tubulin* > *Lbβ-Actin2* > *LbGapdh* > *Lb18S rRNA* [33]. The best reference genes for developmental stages of *S. litura* were *GAPDH* and ubiquinol-cytochrome C reductase (*UCCR*) [30]. In *P. xylostella, EF1* was the optimal reference gene for different development stages [31]. In *S. exigua*, superoxide dismutase (*SOD*), β-actin2 (*ACT2*), *GAPDH, EF1* and β-actin1 (*ACT1*) were stably expressed in samples representing all developmental stages [32]. Our research showed that three genes (*18S rRNA, EF1* and *GAPDH*) comprise the best set of reference genes for different developmental stages and sexes of *S. inferens*.

To validate our findings, we evaluated the expression of *hsp83* in different tissues, developmental stages, sexes, and temperatures. *S. inferens hsp83* is a member of the HSP90 gene family, proteins participate in folding, structural integrity, and the regulation of a subset of cytosolic proteins; furthermore, HSP90 proteins account for 1% of the soluble protein in most tissues, even in the absence of stress [24]. In *S. inferens, hsp83* was consistently expressed in all tissues and developmental stages with the highest expression in eggs. However, when an unstable reference gene was used for qRT-PCR normalization, the profile for *hsp83* expression was significantly altered. Thus, selection of valid, appropriate reference genes is a critical aspect of normalizing target gene expression and interpretation of the data.

The analysis of genes expressed in response to cold treatment may provide future insight into the cold tolerance characteristics and strategy of *S. inferens*. It is interesting to note that this pest has recently dispersed to more northern regions of China. As part of this paper, we evaluated which reference genes were most suitable for normalizing gene expression in *S. inferens* exposed to low temperatures. The reference genes identified in this study will facilitate future studies of the mechanistic basis of cold tolerance in *S. inferens*. Experiments designed to select reference genes appropriate for high temperatures will be conducted in the future, and these will provide some insight into the dispersal and adaptation strategies of this important pest.

## Conclusion

In this study, seven candidate reference genes for normalizing qRT-PCR data in *S. inferens* were evaluated under various experimental conditions. Our results indicate that a suite of internal reference genes are recommended to accurately normalize and quantify gene expression in this important pest of rice. This study establishes standards for gene expression in the *S. inferens*, and it provides a solid foundation for future studies aimed at understanding various gene functions in this insect.

## Supporting Information

S1 Fig Specificity of qRT-PCR amplification. Dissociation curves of eight genes (*18S rRNA* (A), *EF1* (B), *GAPDH* (C), *RPS13* (D), *RPS20* (E), *TUB* (F), *ACTB* (G), and *hsp83* (H)) reveal single peaks.
(TIF)

S1 Table Cycle threshold of *hsp83* in experimental conditions.
(DOCX)
