# Supplementary figures and images for: Exploring Valid Reference Genes for Quantitative Real-Time PCR Analysis in Sesamia inferens (Lepidoptera: Noctuidae)
Source: PLoS One. 2015 Jan 13;10(1):e0115979. doi: 10.1371/journal.pone.0115979 (PMC4293147; doi:10.1371/journal.pone.0115979)

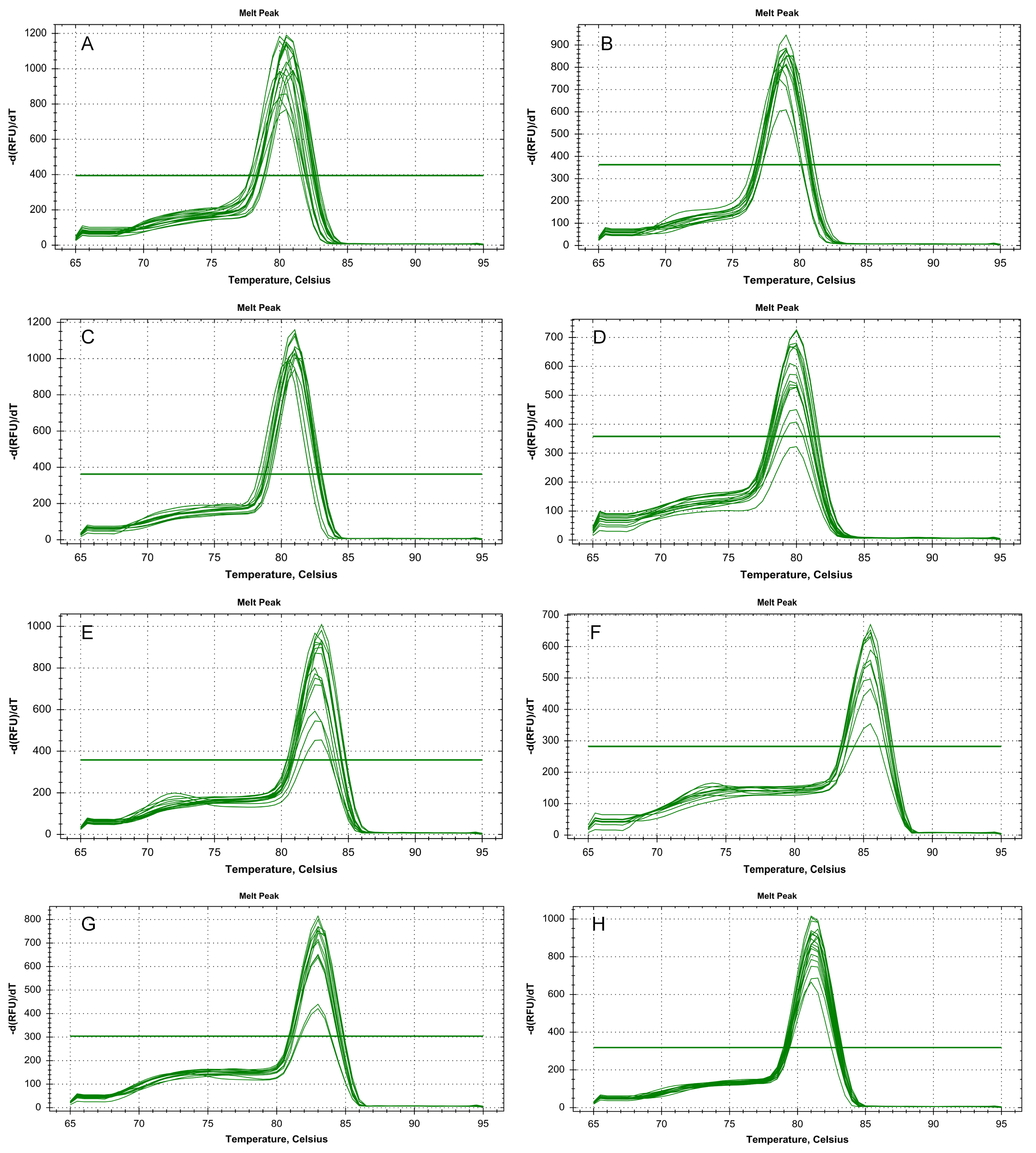

Supplement: S1 Fig — Dissociation curves of eight genes (18S rRNA (A), EF1 (B), GAPDH (C), RPS13 (D), RPS20 (E), TUB (F), ACTB (G), and hsp83 (H)) reveal single peaks. (TIF) [file pone.0115979.s001.tif]
