# Supplementary material for: Exploring Valid Reference Genes for Quantitative Real-Time PCR Analysis in Sesamia inferens (Lepidoptera: Noctuidae)
Source: PLoS One. 2015 Jan 13;10(1):e0115979. doi: 10.1371/journal.pone.0115979 (PMC4293147; doi:10.1371/journal.pone.0115979)
Supplement: S1 Table — Cycle threshold of hsp83 in experimental conditions. (DOCX) [file pone.0115979.s002.docx]

**Table S1. Cycle threshold of *hsp83* in experimental conditions.**

| **Conditions** | | **Cycle threshold (Ct value, mean ± SE)** |
| --- | --- | --- |
| **Tissues** | HE | 26.03 ± 0.30 |
|  | EP | 25.05 ± 0.25 |
|  | FG | 25.78 ± 0.33 |
|  | MG | 25.15 ± 0.17 |
|  | HG | 24.97 ± 0.26 |
|  | HC | 26.15 ± 0.26 |
|  | FB | 26.09 ± 0.08 |
|  | MT | 26.59 ± 0.23 |
|  | SG | 28.77 ± 0.63 |
| **Developmental stages and sex** | E | 21.26 ± 0.15 |
|  | L1 | 25.58 ± 0.16 |
|  | L2 | 24.96 ± 0.45 |
|  | L3 | 24.69 ± 0.53 |
|  | L4 | 23.99 ± 0.08 |
|  | L5 | 24.62 ± 0.07 |
|  | L6 | 24.99 ± 0.35 |
|  | FP | 24.63 ± 0.10 |
|  | MP | 24.67 ± 0.17 |
|  | FA | 25.50 ± 0.24 |
|  | MA | 25.77 ± 0.10 |
| **Temperatures (°C)** | 27 | 25.36 ± 0.66 |
|  | 0 | 23.64 ± 0.05 |
|  | -2 | 22.11 ± 0.02 |
|  | -4 | 22.10 ± 0.15 |
|  | -6 | 22.68 ± 0.20 |
|  | -8 | 20.64 ± 0.07 |

The abbreviations HE, EP, FG, MG, HG, HC, FB, MT, and SG represent heads, epidermis, foregut, midgut, hindgut, haemocytes, fat body, Malpighian tubules, and salivary glands, respectively. And eggs, larvae (first, second, third, fourth, ﬁfth, and sixth instar), female and male pupae, and female and male adults, which are designated E, L1, L2, L3, L4, L5, L6, FP, MP, FA, and MA, respectively.
